# Antibiotic resistance of *Streptococcus pneumoniae*, isolated from nasopharynx of preschool children with acute respiratory tract infection in Lithuania

**DOI:** 10.1186/s12879-016-1544-9

**Published:** 2016-05-20

**Authors:** Indrė Stacevičienė, Sigita Petraitienė, Daiva Vaičiūnienė, Tomas Alasevičius, Jūratė Kirslienė, Vytautas Usonis

**Affiliations:** Clinic of Children’s Diseases, Faculty of Medicine, Vilnius University, Santariskiu Str. 4, Vilnius, Lithuania; Children‘s Hospital, Affiliate of Vilnius University Hospital Santariskiu Klinikos, Santariskiu Str. 7, Vilnius, Lithuania

**Keywords:** *Streptococcus pneumoniae*, Antibiotic resistance, Nasopharyngeal colonization, Preschool children, Respiratory tract infection

## Abstract

**Background:**

Increasing pneumococcal resistance to commonly used antibiotics and multidrug resistance is a serious public health concern. Data on distribution of resistant *Streptococcus pneumoniae* (SPn) strains among children in Lithuania are limited. We evaluated the circulation of SPn serotypes and antimicrobial susceptibility among preschool children in Lithuania before the introduction of universal infant pneumococcal vaccination.

**Methods:**

A prospective study was carried out from February 2012 to March 2013 in five cities of Lithuania. A total of 900 children under six years of age who presented to primary care centre or a hospital emergency department with acute respiratory tract infection were enrolled in the study. Nasopharyngeal swabs were obtained and cultured for SPn. Positive samples (*n* = 367) were serotyped and tested for antimicrobial susceptibility. Associations of pneumococcal non-susceptibility with study site, season, age, sex, attendance of day care centre and treatment with antimicrobials (between one and six months prior the study) were evaluated.

**Results:**

About a half (56.7 %) of SPn strains were susceptible to all the antibiotics tested. Pneumococcal non-susceptibility to penicillin, erythromycin, clindamycin and trimethoprim–sulphamethoxazole was 15.8, 21.3, 16.9 and 27.3 %, respectively. None of the tested isolates was resistant to norfloxacin or vancomycin. We found a geographical variation of pneumococcal resistance within the cities of the country. Age, sex, the attendance of day care centre and treatment with antimicrobials prior the study was not significantly associated with a carriage of non-susceptible SPn strains. Among non-susceptible SPn serotypes 67.9 %–82.4 % were present in currently available pneumococcal conjugate vaccines.

**Conclusions:**

The rates of nasopharyngeal SPn susceptibility to penicillin and macrolides are still high among preschool children in Lithuania, however they are lower compared with previous studies. A strict policy with respect to antibiotic prescription together with widespread use of vaccination could potentially reduce the carriage rate of antibiotic-resistant pneumococci in our country.

## Background

*Streptococcus pneumoniae* (*SPn*) colonises the nasopharynx and it may be asymptomatic or result in various types of diseases including acute otitis media, sinusitis, pneumonia, sepsis and meningitis, which are a considerable burden [1, 2]. World Health Organization (WHO) estimated that at the beginning of infants vaccination with pneumococcal conjugated vaccines (PCV) 14.5 million cases of serious illness and 7 % of all cause-child mortality [3] under five years of age were due to pneumococcal infections. Despite the effectiveness of PCV vaccination in different countries, WHO reports that pneumococcal related deaths among children under five years of age remains high [4]. Increasing pneumococcal resistance to commonly used antibiotics such as macrolides or cephalosporins and multidrug resistance is another serious public health concern [5].

Antibiotic susceptibility of SPn has a large variation among European countries. For example, non-susceptibility to penicillin of non-invasive SPn isolates varies from 1.7 % in Norway [6] to 83 % in Romania [7] and non-susceptibility to erythromycin varies from 1.2 % in the Czech Republic [8] to 65.5 % in Italy [9]. Due to geographical diversity of the resistance of SPn strains dependent on the local antimicrobial policy, epidemiological studies in each geographical region are needed [10].

Data on distribution of resistant SPn strains among children in Lithuania are limited. Only 37 and 59 SPn strains, which caused invasive pneumococcal diseases in children and adults, were tested for antimicrobial susceptibility in our country in 2012 and 2013, respectively [11]. These findings do not necessarily reflect the actual antimicrobial susceptibility of SPn in Lithuania and to the moment there is no official data describing invasive pneumococcal resistance among paediatric patients.

Three studies of SPn nasopharyngeal carriage and antimicrobial susceptibility in healthy children have been performed previously (in 1999, 2001 and 2006) [12, 13], in which a total of 1625 children from the same 13 day-care centres were enrolled. Non-susceptibility to penicillin increased from 6.3 % in 1999 to 9.6 % in 2006. Higher increase of non-susceptibility was observed to erythromycin: 4.7 in 1999 and 9.6 % in 2006. These studies were rather limited because all of them were performed during a short period of time (in February and March) and the data presented only one city of the country (Vilnius).

Our study performed in 2012–2013 evaluated the circulation of SPn serotypes and antimicrobial susceptibility among children with acute respiratory tract infection (RTI) under six years of age in Lithuania before the introduction of universal infants PCV vaccination in the country in October 2014 [14]. At the time of the study two PCVs (10-valent (PCV-10) and 13-valent (PCV-13)) were available only on private market. PCV vaccination coverage was unknown but probably it was low as vaccination costs were relatively high and not reimbursed. Results of this study might be the background for further investigations on the impact of PCV vaccination on the distribution of resistant SPn serotypes in Lithuania, including the widely discussed phenomenon of replacement in circulating resistant SPn serotypes due to vaccination [15]. SPn colonisation rate, serotype distribution and the influence on the clinical outcome were published separately [16, 17].

## Methods

This prospective study was carried out from February 2012 to March 2013. Eight primary care centres (PCC) in Lithuania’s five cities (Vilnius (*n* = 2), Kaunas (*n* = 2), Klaipeda (*n* = 2), Panevezys (*n* = 1), Alytus (*n* = 1)) from all main regions of the country and emergency department (ED) of Children’s Hospital, Affiliate of Vilnius University Hospital Santariskiu Klinikos in Vilnius were involved in examining children for SPn nasopharyngeal carriage and antimicrobial susceptibility.

Children under six years old, who visited a primary care physician or a paediatrician because of either upper or lower acute RTI, were enrolled into the study. The main symptoms of acute RTI were acute onset, fever (37.2 °C or higher), runny nose, sneezing, cough and sore throat. Children were excluded if another cause of fever was identified (e.g., confirmed urinary tract infection, etc.). Children were not included if they had been vaccinated with any pneumococcal vaccine (because of the vaccine’s effect on reducing colonization with vaccine serotypes [18]) or had taken antibiotics within one month prior the enrolment (as this can diminish the yield of nasopharyngeal culture samples [19]).

Nasopharyngeal swabs were taken at the time of enrolment in the study using Culturette with Amies transport medium (Deltalab, Spain) and transported to a certified laboratory of Children’s Hospital, Affiliate of Vilnius University Hospital Santariskiu Klinikos in Vilnius within 48 h from collection. Hare et al. compared several methods for nasopharyngeal samples transport and found Amies transport medium adequate for SPn detection [20]. However, according WHO skim milk-tryptone-glucose-glycerol (STGG) remains the medium of choice for transport and storage of nasopharyngeal swabs [21].

Classic cultural methods (cultivation in 5 % CO_2_, colony morphology, Gram staining, catalase test, optochin sensitivity) were used to isolate SPn from the swabs [21, 22]. All the isolates were sensitive to optochin. Bacterial antigen rapid latex agglutination test (*Wellcogen*, Remel Europe Limited, United Kingdom) was used for the confirmation. Serotypes were determined by means of latex agglutination reaction using the Pneumotest-Latex kit and selected Latex Factor sera: 6b, 6c, 7b, 9 g, 18c, 18f, 19b, 19c and 23b (Statens Serum Institut, Copenhagen, Denmark).

SPn susceptibility to penicillin was determined by disk-diffusion method using 1 μg oxacillin disk (*Bio*-*Rad*, France). Isolates showing inhibition zones ≤19 mm were confirmed by the penicillin Etest (*bioMerieux*, France). Breakpoints of minimal inhibitory concentrations (MICs) were interpreted according to the European Committee on Antimicrobial Susceptibility Testing recommendations (EUCAST, 2012) [23]. SPn strains were defined as penicillin susceptible SPn (PSSP, MIC ≤0.06 mg/l), penicillin intermediate SPn (PISP, MIC >0.06–2.0 mg/l) and penicillin resistant SPn (PRSP, MIC >2 mg/l). PISP and PRSP were grouped as penicillin non-susceptible SPn (PNSP).

Susceptibility to erythromycin, clindamycin, trimethoprim–sulphamethoxazole, norfloxacin and vancomycin was determined by disk-diffusion method using Mueller–Hinton agar, containing horse blood 5 % and 20 mg/l ß-nicotinamide adenine dinucleotide (*Bio*-*Rad*, France). Inoculated plates were incubated at 37 °C in 5 % CO_2_. Results were interpreted according to EUCAST, 2012 [23]. Multidrug resistance (MDR) was defined as non-susceptibility to penicillin and ≥2 other non–β-lactam antimicrobial classes [24].

### Statistical analysis

The data were analysed using SPSS (*Statistical Package for the Social Sciences*) software 16. Multivariable Poisson regression with robust variance estimation was used to assess the association of study site (PCCs of Kaunas, Klaipeda, Panevezys vs Vilnius ED and PCC), season (spring, autumn, winter vs summer), age (24–47 and 48–71 vs 0–23 months), sex (male vs female) and attendance of day care centre (DCC; non-attending vs attending) with SPn non-susceptibility. Univariable Poisson regression with robust parameter estimates was used to analyse the associations of pneumococcal non-susceptibility to separate antibiotics with sex, study site, and treatment with antimicrobials (between one and six months prior the study). Cross tabulation with chi-squared test was used to test statistical significance for differences between two groups. Statistical significance was defined by *p* < 0.05.

## Results

The data collected from the 900 study participants during the one-year study period were analysed: 636 patients at the PCCs and 264 at the hospital ED. The enrolled children comprised 408 girls and 492 boys under six years of age with acute RTI. The distribution of enrolled children by sex and age was similar in all the cities, with the exception of Alytus, which may be due to a very small number of study participants in this city (*n* = 18) [16].

A total of 367 SPn strains (one per patient) were isolated from the 900 samples collected, giving a colonisation rate of 40.8 %. A detailed analysis of SPn colonisation and serotype distribution in relation to various factors were published separately [16, 17].

All isolated SPn strains (*n* = 367) were tested for antimicrobial susceptibility. About a half (56.7 %, *n* = 208) of SPn strains were susceptible to all the antibiotics tested and 76 % (*n* = 279) were susceptible to penicillin and macrolides. A single drug resistance was observed in 24.0 % (*n* = 88) of SPn isolates with a predominance of non-susceptibility to trimethoprim-sulphamethoxazole (19.3 %, *n* = 71). Dual resistance was present in 5.2 (*n* = 19) and MDR - in 12.5 % (*n* = 46) of SPn isolates.

The highest penicillin MIC was 2 mg/l. Hence, according to the 2012 EUCAST breakpoints [23], none of the strains fell into the resistant category, but 15.8 % (*n* = 58) were PISP. Only 12.1 % (*n* = 7) of PISP were susceptible to other antibiotics tested while other PISP were concomitantly non-susceptible to erythromycin (82.8 %, *n* = 48), clindamycin (77.6 %, *n* = 45) or trimethoprim-sulphamethoxazole (34.5 %, *n* = 20). In addition, 8.6 (*n* = 5) of PISP showed dual resistance, while 51.7 (*n* = 30) and 27.6 % (*n* = 16) were resistant to 3 or 4 antibiotics tested, respectively.

SPn resistance to clindamycin was 16.9 % (*n* = 62). Higher rates of resistance were present to erythromycin: 21.0 (*n* = 77) were resistant and 0.3 % (*n* = 1) was intermediately susceptible. The highest non-susceptibility rates of pneumococci were found to trimethoprim-sulphamethoxazole, whereas 21.0 (*n* = 77) were resistant and 6.3 % (*n* = 23) intermediately susceptible. None of the tested isolates was resistant to norfloxacin and vancomycin.

The rate of SPn antimicrobial non-susceptibility in relation to various factors is summarized in Table 1. Antibiotic non-susceptibility of SPn was similar at ED compared with PCCs, 44.6 (54/121) and 42.7 % (105/246) respectively (PR (95 % CI) – 1.035 (0.854–1.255), *p* = 0.724). A detailed analysis revealed a higher prevalence of PISP and MDR SPn at PCCs compared with ED (19.1 % *vs* 9.1 %, PR (95 % CI) – 2.102 (1.131–3.904), *p* = 0.019 for PISP and 15.4 % vs 6.6 %, PR (95 % CI) – 2.336 (1.125–4.852) *p* = 0.023 for MDR SPn, respectively). Differences of SPn susceptibility to erythromycin, clindamycin and trimethoprim-sulphamethoxazole among those isolated from patients at PCCs and the ED of the hospital were not statistically significant (*p* = 0.641, *p* = 0.188 and *p* = 0.613, respectively).

**Table 1:** *Streptococcus pneumoniae* non-suscebtibility in relation to various factors

Characteristic		Susceptible isolates (*n* = 208), n (%)	Non-susceptible isolates (*n* = 159), n (%)	Total (*n* = 367)	PR (95 % CI)	*P* value
Age (months)						
0–23^b^		49 (53.3)	43 (46.7)	92		
24–47		117 (58.8)	82 (41.2)	199	0.953 (0.858–1.059)	0.370
48–71		42 (55.3)	34 (44.7)	76	0.992 (0.874–1.125)	0.895
Sex						
Male		114 (56.4)	88 (43.6)	202	1.006 (0.937–1.081)	0.864
Female^b^		94 (57.0)	71 (43.0)	165		
Day care centre attendance						
Attending^b^		153 (56.7)	117 (43.3)	270		
Non-attending		53 (56.4)	41 (43.6)	94	1.013 (0.912–1.126)	0.808
Season						
Spring		72 (52.9)	64 (47.1)	136	1.181 (1.024–1.363)	0.022
Summer^b^		23 (71.9)	9 (28.1)	32		
Autumn		78 (59.1)	54 (40.9)	132	1.097 (0.947–1.270)	0.219
Winter		35 (52.2)	32 (47.8)	67	1.154 (0.990–1.344)	0.067
Cities of Lithuania						
Vilnius	PCC	45 (52.3)	41 (47.7)	86		
	ED	67 (55.4)	54 (44.6)	121		
	Total^b^	112 (54.1)	95 (45.9)	207		
Kaunas	PCC	31 (59.6)	21 (40.4)	52	0.939 (0.843–1.046)	0.253
Klaipeda	PCC	17 (70.8)	7 (29.2)	24	0.870 (0.733–1.033)	0.112
Panevezys	PCC	47 (57.3)	35 (42.7)	82	0.985 (0.891–1.090)	0.774
Alytus^a^	PCC	1 (50.0)	1 (50.0)	2		

*PCC* primary care centre, *ED* the emergency department of Children’s Hospital, Affiliate of Vilnius University Hospital Santariskiu Klinikos, Vilnius. PR (95 % CI) - prevalence ratio and 95 % confidence interval of Streptococcus pneumoniae non-susceptibility to one or more antibiotics tested (penicillin, erythromycin, clindamycin, trimethoprim–sulphamethoxazole, norfloxacin and vancomycin), using Multivariable Poisson regression

^a^Note that Alytus was excluded from this comparison because of the small number of *S. pneumoniae* isolates (*n* = 2)

^b^Reference group for each comparison

**Fig. 1:**
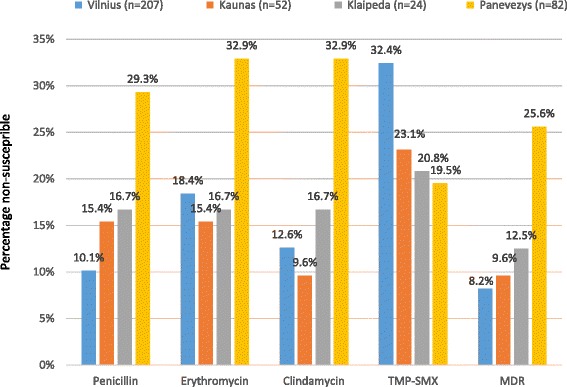
Distribution of non-susceptible *Streptococcus pneumoniae* nasopharyngeal strains in the study sites of Lithuania. *TMP-SMX - trimethoprim–sulphamethoxazole; MDR - multidrug resistance. Study sites were primary care centres of Vilnius, Kaunas, Klaipeda, Panevezys and Alytus and the emergency department of Children’s Hospital, Affiliate of Vilnius University Hospital Santariskiu Klinikos in Vilnius. Note that Alytus was excluded from this comparison because of the small number of S. pneumoniae isolates (n = 2). Using univariable Poisson regression analysis, significant differences were found in these comparisons: non-susceptibility to penicillin was higher in Panevezys vs Vilnius (PR: 2.885, p = 0.000), non-susceptibility to erythromycin was higher in Panevezys vs Vilnius (PR: 1.794, p = 0.007) and Kaunas (PR: 2.140, p = 0.035), resistance to clindamycin was higher in Panevezys vs Vilnius (PR: 2.621, p = 0.000) and Kaunas (PR: 3.424, p = 0.007), non-susceptibility to TMP-SMX was higher in Vilnius vs Panevezys (PR: 1.659, p = 0.039) and MDR was higher in Panevezys vs Vilnius (PR: 3.118, p = 0.000) and Kaunas (PR: 2.663, p = 0.035)*

Antimicrobial non-susceptibility of SPn varied in different studied sites of Lithuania: the highest rates were found in Vilnius and the lowest – in Klaipeda (Table 1). Higher rates of MDR and non-susceptibility to penicillin, erythromycin and clindamycin were found in Panevezys while non-susceptibility to trimethoprim-sulphamethoxazole was more common in Vilnius (Fig. 1). Alytus was excluded from this comparison because of the small number of SPn isolates (*n* = 18). Sex and age were not significantly associated with carriage of non-susceptible SPn strains (Table 1) but there was a tendency of higher non-susceptibility to erythromycin (25.5 % vs 17.8 %, PR (95 % CI) - 1.428 (0.962–2.120), *p* = 0.077) and clindamycin (20.0 % vs 14.4 %, PR (95 % CI) - 1.393 (0.884–2.195), *p* = 0.153) in girls compared with boys. SPn non-susceptibility to different antibiotics was similar among age groups (Table 2). The attendance of DCC had no significant effect to the susceptibility of pneumococci (Table 1).

**Table 2:** Distribution of *Streptococcus pneumoniae* antimicrobial non-susceptibility among age groups

Age groups in months	Non-susceptibility (ns) or resistance (r) to antibiotics, n (%)				MDR, n (%)
	Penicillin (ns)	Erythromycin (ns)	Clindamycin (r)	TMP-SMX (ns)	
0–23^a^ (*n* = 92)	18 (19.6)	21 (22.8)	19 (20.7)	25 (27.2)	14 (15.2)
24–47 (*n* = 199)	27 (13.6)	40 (20.1)	31 (15.6)	53 (26.6)	23 (11.6)
P value	0.186	0.593	0.283	0.923	0.382
48–71 (*n* = 76)	13 (17.1)	17 (22.4)	12 (15.8)	22 (28.9)	9 (11.8)
*P* value	0.683	0.944	0.422	0.799	0.529
Total	58 (15.8)	78 (21.3)	62 (16.9)	100 (27.3)	46 (12.5)

*TMP-SMX* trimethoprim–sulphamethoxazole, *MDR* multidrug resistance

^a^Reference group for each comparison (chi-squared test)

A significant decrease of non-susceptibility to all tested antibiotics was observed in summer compared to spring (Table 1), but the differences to separate antibiotics were not statistically significant. The rate of susceptible SPn strains did not differ significantly between children who were treated with antimicrobials during the period between one and six months prior the nasopharyngeal sample and children who were not (52.0 % (51/98) vs 57.4 % (143/249), PR (95 % CI) - 0.906 (0.729–1.127), *p* = 0.376).

**Fig. 2:**
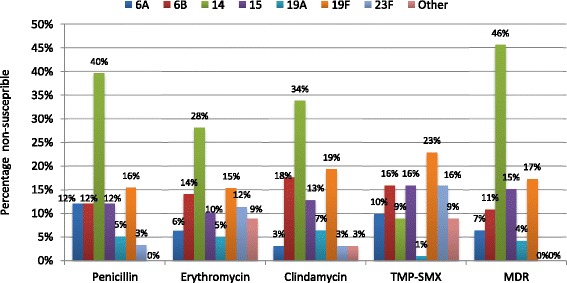
The most common serotypes of non-susceptible *Streptococcus pneumoniae* nasopharyngeal strains. *TMP-SMX - trimethoprim–sulphamethoxazole; MDR - multidrug resistance*

The most common serotypes of non-susceptible SPn strains were 19 F (20.8, 33/159), 14 (15.7, 25/159), 6B (14.5, 23/159), 15 (13.8, 22/159) and 23 F (13.8 %, 22/159). Serotype distribution of SPn strains non-susceptible to different antibiotics are shown in Fig. 2. Among non-susceptible serotypes 67.9 (108/159) were present in PCV10 (serotypes 1, 4, 5, 6B, 7 F, 9 V, 14, 18C, 19 F and 23 F) and 82.4 % (131/159) in PCV13 (serotypes 1, 3, 4, 5, 6A, 6B, 7 F, 9 V, 14, 18C, 19A, 19 F and 23 F). Among MDR serotypes 73.9 (34/46) were present in PCV10 and 84.8 % (39/46) in PCV13.

## Discussion

### Susceptibility of non-invasive *S. pneumoniae*

The data presented illustrate the resistance of non-invasive SPn to antimicrobials among preschool children before the introduction of universal infants PCV vaccination.

The rate of PSSP was quite high (84.2 %) and there were no PRSP strains found in the nasopharynx. Similar results were observed among invasive SPn isolates from children and adults in our country during the study years: 83.8 in 2012 and 76.3 % in 2013 [11]. However, our findings indicates lower PSSP rates than those in healthy children aged 2–7 years in the previous nasopharyngeal carriage studies in Lithuania: 93.8 in 1999, 90.0 in 2001 and 90.4 % in 2006 [13].

A wide geographical variation exists in the prevalence of PNSP nasopharyngeal carriage in Europe. The rates of PNSP seen in our study (15.8 %) was higher to those observed in healthy children in Norway (1.7) [6], the Netherlands (2.7) [25], the Czech Republic (3) [8] and Estonia (6 %) [26] before the widespread use of PCV vaccination. Contrarily, results of pre-vaccination studies conducted in Greece [27], Poland [28] and Romania [7] have shown higher rates of PNSP in healthy children: 34.7, 39.2 and 83 %, respectively. A decrease of resistance to penicillin was reported due to the PCV vaccination [29, 30].

According to our results, SPn susceptibility to erythromycin and clindamycin was also high (78,7 and 83.1 %, respectively). However, previously reported rates of nasopharyngeal SPn susceptibility to erythromycin and clindamycin were even higher among healthy preschool children in Vilnius (90.4 and 98 % in 2006, respectively) [13]. A significant decrease (from 100 % to 82,1 %) of resistance to macrolides of invasive SPn isolates was also observed during the period 2006–2013 in Lithuania [31]. It may be due to an increased use of macrolides in the clinical practice.

Non-susceptibility of non-invasive SPn strains to erythromycin and clindamycin varies from 1.2 [8] to 65.5 [9] and from 0.6 % [8] to 49 % [32] in different European countries, respectively. Our results are similar to those observed among non-invasive SPn strains in paediatric population in Poland (29.5 and 29.2 %, respectively) [28] and Russia (16.7 and 19.3 %, respectively) [33] before routine PCV vaccination in these countries.

High levels of SPn resistance to trimethoprim-sulphamethoxazole were observed previously among non-invasive strains in healthy preschool children in Lithuania [13]. Trimethoprim-sulphamethoxazole is not widely used to treat respiratory tract infections in children, therefore the non-susceptibility to trimethoprim-sulphamethoxazole decreased from 60.0 % in 1999 to 46.0 % in 2006 [13] and it was 27.3 % in the current study. Pneumococcal resistance to trimethoprim-sulphamethoxazole was found to be high up to 66–67 % in Estonia [26] and Romania [7] while the rates were lower in Norway (4.6) [6], the Netherlands (12.9) [25] and the Czech Republic (15.7 %) [8].

Our data suggest that multidrug resistance of non-invasive SPn among children in Lithuania (12.5 %) is intermediate as compared with other European countries in the pre-PCV-vaccination era. Higher rates of MDR strains were found in Greece (25) [27], Poland (39.5) [28] and Romania (67 %) [7], while lower rates were observed in Norway (4.5) [6], Estonia (4) [26] and the Netherlands (1.9 %) [25]. A decrease of resistance to macrolides, trimethoprim-sulphamethoxazole and MDR was reported due to the PCV vaccination [6, 30]. Resistance of SPn varies not only among the countries. We found a geographical variation of SPn resistance within the cities of the country; therefore the attention should be paid to the use of penicillin and macrolides in Panevezys and to the use of trimethoprim-sulphamethoxazole in Vilnius.

It is important to note that most studies have focused on SPn carriage in healthy children while in our study children with acute RTI were enrolled. Similarly, Mayanskiy N et al. examined antibiotic susceptibility of non-invasive SPn in children aged under six years with symptoms of an acute RTI and with chronic lung disease in Moscow, Russia. Non-susceptibility to penicillin, erythromycin and MDR was found in 28, 26 and 22 % of pneumococcal strains, respectively [34]. We found a bit lower rates of SPn resistance and it may be due to the enrolment of previous healthy children or the differences in antibiotic policy.

#### Serotype distribution of resistant *S. pneumoniae* strains

Serotype distribution of resistant SPn strains has been changed during different study years in Lithuania. Serotypes 6B, 9 V and 23 F were prevalent among PNSP strains in 2006 [13], while serogroups/serotypes 14, 19 F, 6A, 6B and 15 were the most common in the current study. Serotypes 23 F and 6B dominated among SPn strains non-susceptible to erythromycin in 2006 [13] and serogroup 14, serotypes 19 F and 6B were the most common according to our data. It is important to note, however, that the study sites and the type of children studied differed, which limit the comparison.

Serogroups 6, 9, 14, 19 and 23 accounted for most drug-resistant SPn before widespread use of PCV in the USA and European countries [7, 27, 35–39]. Our findings are in accordance with this data with an exception of serogroup 9, which was uncommon in our study, while serogroup 15 was more prevalent. The introduction and widespread use of PCVs have changed the situation in many countries. For example, non-invasive PNSP isolates were mostly represented by serotypes 14, 23 F, 19 F, 6B, 9 V, 6A and 19A in France, in 2001/2002. By contrast, serotypes 19A, 19 F and non-PCV serotypes, particularly serotypes 11A, 15A, 15B/C, 29 and 35B were dominating after widespread use of PCV vaccination in 2013/2014 [35]. The previously highly prevalent resistant serotypes seem to be successfully suppressed, and the emergence of new – often less resistant, but sometimes more virulent and invasive – serotypes is observed [39].

The data suggest that vaccination could potentially reduce the carriage rate of antibiotic-resistant pneumococci in Lithuania as a majority (67.9 %–82.4 %) of non-susceptible SPn serotypes belonged to serotypes included in PCVs. Our study was performed before the implementation of the universal programme of PCV vaccination in Lithuania, therefore it provides a basis for future comparisons of resistant SPn carriage and serotype distribution between the pre- and post-vaccination era in the country. It would be also helpful in improving antimicrobial policy in Lithuania.

## Conclusions

The rates of nasopharyngeal SPn susceptibility to penicillin and macrolides are high among preschool children in Lithuania, however they are lower compared with previous studies. A strict policy with respect to antibiotic prescription together with widespread use of vaccination could potentially reduce the carriage rate of antibiotic-resistant pneumococci in our country.

## Abbreviations

DCC: day care centre
ED: emergency department
EUCAST: European Committee on Antimicrobial Susceptibility Testing
MDR: multidrug resistance
MIC: minimal inhibitory concentration
PCC: primary care centres
PCV: pneumococcal conjugate vaccine
PISP: penicillin intermediate *Streptococcus pneumoniae*
PNSP: penicillin non-susceptible *Streptococcus pneumoniae*
PRSP: penicillin resistant *Streptococcus pneumoniae*
PSSP: penicillin susceptible *Streptococcus pneumoniae*
RTI: upper respiratory tract infection
SPn: *Streptococcus pneumoniae*
SPSS: Statistical Package for the Social Sciences
WHO: World Health Organization
